# Polycystic Ovary Syndrome Detection Machine Learning Model Based on Optimized Feature Selection and Explainable Artificial Intelligence

**DOI:** 10.3390/diagnostics13081506

**Published:** 2023-04-21

**Authors:** Hela Elmannai, Nora El-Rashidy, Ibrahim Mashal, Manal Abdullah Alohali, Sara Farag, Shaker El-Sappagh, Hager Saleh

**Affiliations:** 1 Department of Information Technology, College of Computer and Information Sciences, Princess Nourah bint Abdulrahman University, P.O. Box 84428, Riyadh 11671, Saudi Arabia; 2 Machine Learning and Information Retrieval Department, Faculty of Artificial Intelligence, Kafrelsheiksh University, Kafrelsheiksh 13518, Egypt; 3 Faculty of Information Technology, Applied Science Private University, Amman 11937, Jordan; 4 Department of Information Systems, College of Computer and Information Sciences, Princess Nourah bint Abdulrahman University, P.O. Box 84428, Riyadh 11671, Saudi Arabia; 5 Faculty of Computers and Informations, South Valley University, Qena 83523, Egypt; 6 Faculty of Computer Science and Engineering, Galala University, Suez 435611, Egypt; 7 Information Systems Department, Faculty of Computers and Artificial Intelligence, Benha University, Banha 13518, Egypt; 8 Faculty of Computers and Artificial Intelligence, South Valley University, Hurghada 84511, Egypt

**Keywords:** polycystic ovary syndrome, machine learning, explainable machine learning, ensemble learning

## Abstract

Polycystic ovary syndrome (PCOS) has been classified as a severe health problem common among women globally. Early detection and treatment of PCOS reduce the possibility of long-term complications, such as increasing the chances of developing type 2 diabetes and gestational diabetes. Therefore, effective and early PCOS diagnosis will help the healthcare systems to reduce the disease’s problems and complications. Machine learning (ML) and ensemble learning have recently shown promising results in medical diagnostics. The main goal of our research is to provide model explanations to ensure efficiency, effectiveness, and trust in the developed model through local and global explanations. Feature selection methods with different types of ML models (logistic regression (LR), random forest (RF), decision tree (DT), naive Bayes (NB), support vector machine (SVM), k-nearest neighbor (KNN), xgboost, and Adaboost algorithm to get optimal feature selection and best model. Stacking ML models that combine the best base ML models with meta-learner are proposed to improve performance. Bayesian optimization is used to optimize ML models. Combining SMOTE (Synthetic Minority Oversampling Techniques) and ENN (Edited Nearest Neighbour) solves the class imbalance. The experimental results were made using a benchmark PCOS dataset with two ratios splitting 70:30 and 80:20. The result showed that the Stacking ML with REF feature selection recorded the highest accuracy at 100 compared to other models.

## 1. Introduction

Polycystic ovary syndrome (PCOS) affects pregnant women and current mothers. PCOS affects the health of women by causing hormonal imbalances and metabolism problems. It is a disease that primarily affects women’s fertility, as 5 to 10% of females suffer from this disease in their childbearing years (15–45) [1]. It is a hormonal disorder that causes problems with the ovaries. In the normal state, the ovaries produce hormones (chemicals that control the functions of the body), namely estrogen (female hormone) and androgens (male hormones), for normal health [2]. In affected women, the hormones are imbalanced, with higher androgens or less estrogen than normal. This causes lumps (fluid-filled sacs) to grow on the ovaries. These lumps gradually enlarge and then obstruct the ovulation process. This disruption of ovulation in women with PCOS reduces their chances of becoming pregnant [3]. Women with PCOS are more likely to develop diabetes, heart disease, high blood pressure, endometrial thickness, sleep apnea, depression, anxiety, eating disorders, and endometrial cancer [4]. In addition to genetic factors, environmental factors may also contribute to PCOS development. In addition to early diagnosis, treatment, and weight loss, long-term complications can be reduced [5].

Artificial intelligence (AI) has revolutionized the detection and treatment of diseases, specifically PCOS [6]. AI-based technologies such as machine learning (ML) algorithms and deep learning networks (DL) have enabled the development of automated systems for the accurate and reliable detection of heart disease [7,8]. AI-based methods can identify patterns in medical data, such as hormone levels, to distinguish PCOS patients from those without the disorder. This improved accuracy could lead to earlier, more accurate diagnoses and better overall outcomes for PCOS patients. Furthermore, AI-based systems can be used to monitor patients over time, providing clinicians with valuable insights into potential treatments and enabling more precise interventions. In short, AI-driven technology has the potential to revolutionize PCOS detection and treatment, providing more effective and efficient care for those suffering from the condition.

Feature selection [9] reduces the number of input variables when developing a predictive model. The goal of feature selection approaches in ML [10] is to find the best features to build effective models of the studied phenomena. It involves automatically selecting features for your ML model pertinent to the problem you are attempting to solve. We accomplish this by adding or removing significant features without altering them. It assists in minimizing the amount of noise in our data and the quantity of our input data. A hybrid model combines two or more different models or strategies to address a challenge or accomplish a goal [11]. A hybrid model can be used in machine learning to combine various algorithms or strategies to increase a model’s performance and accuracy [12]. For instance, a hybrid model can improve both the accuracy and efficiency of neural networks and decision trees by combining both qualities. Combining statistical and rule-based models is another illustration of a hybrid model.

In ML, there is always a tradeoff between the complexity and performance of the developed model. A simple model (i.e., linear regression) could be more interoperable and provide a more understandable explanation than complex ML and DL models [13]. Therefore, providing a clear explanation of such a complex model is a significant point in increasing trust in the developed model. Explainability is motivated by the lack of model transparency of complex (black-box) models that lack model trust [14]. Explainable AI (XAI) techniques improve model predictions’ understanding, interpretability, and reliability. Explainability has two primary levels, i.e., local explainability and global explainability. Global explainability explains the final decision at the level of all data points. It provides casual analysis in terms of global fidelity. It only explained the instance level with the importance of such a level [15]. Local allegiance could explain in terms of all samples. It provides a more accurate explanation.

Our research aims to provide model explanations to ensure efficiency, effectiveness, and trust in the developed model through local and global explanations. Feature selection methods with different types of ML models and proposed are proposed to predict PCOS. The following are the main contributions of the suggested work:

- A combination of SMOTE (Synthetic Minority Oversampling Techniques) and ENN (Edited Nearest Neighbour) solves the class imbalance.
- Applying feature selection (FS) to reduce data dimensionality and select the optimal feature set.
- Applying Bayesian Optimization with cross-validation to optimize ML algorithms and enhance accuracy.
- Proposing stacking ML and comparing it with different ML models using evaluation methods, including accuracy (Acc), precision (P), recall (R), F1 score (F1), and area under the receiver operating characteristic (ROC) (AUC) curve.
- Increasing the model trust by clearly explaining the final prediction using global and local explainability terms.

This paper is organized as follows, Section 2 summarizes the related work in the PCOS domain. The dataset and proposed methodology can be described in Section 3. Section 4 shows the results. Section 5 shows the discussion, including a comparison with related work and model explainability. The paper concludes in Section 6.

## 2. Related Work

The authors applied ML models to PCOS from Kaggle to predict PCOS. For example, in [16], the authors applied gradient boosting, RF, LR, and a hybrid RFLR model that integrated RF with LR with a univariate feature selection (UFS) algorithm from the PCOS dataset. They split the dataset using holdout and cross-validation methods to train and test models. The result showed that RFLR with UFS achieved the highest performance.

In [17], the authors reduced the number of features using Principal Component Analysis (PCA). They applied NB, KNN, LR, RF, and SVM with selected features to predict PCOS. The result showed that RF achieved the highest accuracy. In [6], the authors used correlation feature selection methodology to select a subset of features from the database. They applied different ML models: SVM, LR, RF, DT, KNN, Quadratic Discriminant Analysis (QDA), Linear Discriminant Analysis (LDA), GB, AdaBoost (AB), XGBoost (XB), and CatBoost, and obtained the optimal model based on correlation thresholds. The result showed that RF was the optimal model.

In [18], the authors compared different models, i.e., CNN, ANN, SVM, DT, and KNN, and applied feature selection methods to diagnose PCOS. RF achieved the best-performing model. In [19], the authors utilized Pearson correlation to determine the best features. The applied SVM, RF, and XG boost multi-layer perceptron with selected features to detect the accuracy rate of their SVM have the highest rate. In [20], the authors proposed a hybrid feature selection approach using filters and wrappers to reduce the number of features. Furthermore, they applied different ML models with selected features to predict PCOS. SVM achieved the highest accuracy.

In [21], they applied SVM, LR, NB, and KNN to detect whether a woman was suffering from PCOS. They used chi-square feature selection methods to select the top 30 features. The accuracy of RF has achieved the highest rate. In [16], the authors used RF, DT, SVM, LR, KNN, XGBRF, and CatBoost Classifier to detect whether a woman was suffering from PCOS. The result showed that CatBoost recorded the highest accuracy.

In [22], the authors used Gini importance to select features. They applied different ML models: KNN, DT, SVM, LR, and NB, to detect PCOS. Based on the accuracy, DT recorded the highest rate. In [23], the authors applied CatBoost, RF, LR, NB, DT, SVM, and DT. Furthermore, they compared their outcomes in terms of the evaluation matrix. CatBoost has the highest accuracy in predicting whether a woman should seek medical help for PCOS. In [24], the authors applied Chi-Square, ANOVA, and Mutual Information to identify insignificant features from the data. They used selected features to detect PCOS by applying SVM, LR, DT, NB, XGBRF, RF, and CatBoost. The CatBoost classifier performed with the best accuracy.

In [25], the authors used ML models: LR, DT, RF, SVM, NB, KNN, AdaBoost, XGBoost, and Extratrees and DL and proposed multi-stacking ML to predict PCOS. They used Explainable AI (XAI) techniques to make model predictions understandable, interpretable, and trustworthy. The result showed that multi-stacking ML recorded the best performance.

## 3. Methodology

**Figure 1:**
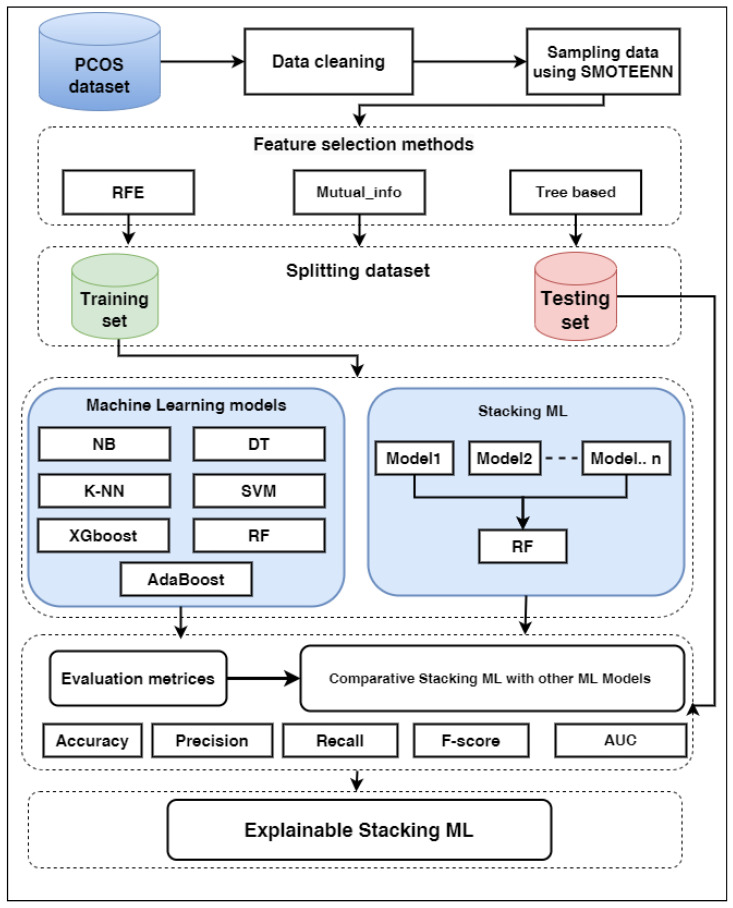
The phases of prediction PCOS.

We applied different ML models: SVM, NB, LR, KNN, RF, DT, XGboost, and AdaBoost, with FS methods to predict PCOS. We proposed Stacking ML models that combine the best ML models. Figure 1 shows the phases of prediction PCOS.

### 3.1. Database Description

We used the PCOS dataset from Kaggle [26], which includes 541 instances and 41 attributes. There are 178 instances of the positive class (1) and 363 instances of the negative class (3). The dataset has a mismatched distribution of classes. The dataset includes two files—we merged two files: PCOS_infertility and PCOS_data_without_infertility, and deleted redundant columns. Table 1 shows the details of the database features.

### 3.2. Data Processing

This stage aims to enhance the quality of the utilized dataset, as it include several missing values and outliers. Medical datasets commonly suffer from such issues due to various causes, including device failure, network loss, irregular time recording, etc. Unfortunately, Several ML models are sensitive to outliers; most cannot handle missing values. Data preprocessing include filling in missing data and data encoding.

#### Filling Missing Values

Many statistical approaches exist to deal with missing data, but it mainly depends on how much data are missing and the importance of the feature missing [27]. When the fraction of the missing data is between 5% and 10%, traditional statistical approaches, such as mean, max, and mode, work exceptionally well. When the fraction of missing values is 20–50%, sophisticated approaches, such as hot-deck [28] and expectation maximization [29], are appropriate. To ensure data reliability in our used data, we choose to remove features with more than 30% missing values. Features with missing values that are less than 30% are imputed using feature means. Remove columns that include many null values: BMI, FSH/LH, and Waist:Hip Ratio. Furthermore, we drop Sl. No, Patient File No. Columns. Filling NA values with the median of that feature: Marraige Status (Yrs), II beta-HCG (mIU/mL), AMH (ng/mL), and Fast food (Y/N).

### 3.3. Data Encoding

Categorical and numeric features are combined in the utilized dataset. Numeric features perform better with ML and DL than categorical ones, unfortunately. Therefore, we encoded all categorical features using the label encoder module of the Scikit-learn library.

### 3.4. Sampling Data

We used SMOTEENN to re-sampling data. The SMOTE-ENN method combines the SMOTE and ENN techniques. SMOTE is an oversampling method, and ENN is an edited closest neighbor undersampling method (ENN). In the ENN approach, the observation and its KNN are removed from the dataset if the majority class of the observation’s KNN and the observation’s class are different. Due to this, information about the minority class in the majority class is lost. By doing this, the bias towards the majority class is lessened, which enhances the performance of machine learning models [30].

### 3.5. Feature Selection Techniques

**Figure 2:**
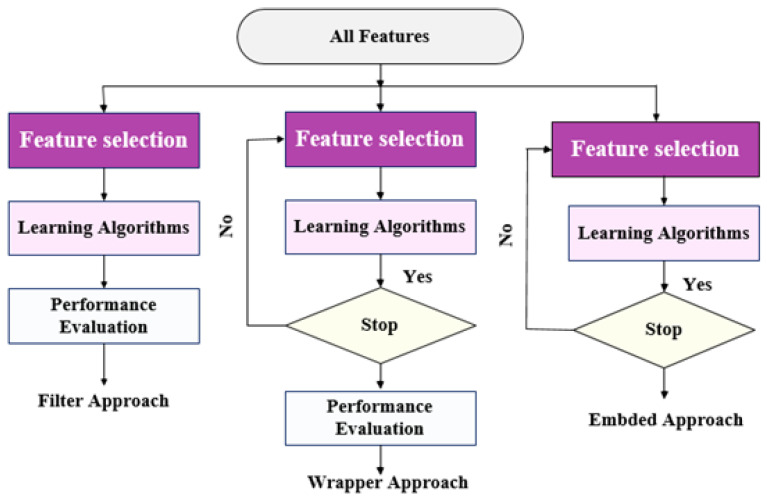
The different types of feature selection methods.

An optimal feature subset is determined by feature selection (FS), which removes irrelevant features to increase learning accuracy [31,32]. The feature subset is chosen from the original feature set based on feature relevance and redundancy. As shown in Figure 2, FS is categorized into three main types according to the interaction with the utilized model: filter approach, wrapper approach, and embedded approach. The following subsection details the different approaches of FS. Our study used one method of each type, such as mutual information-based, REF, and tree based (RF).

#### 3.5.1. Filter Approach

The filter approach utilizes statistical tests to score all features and select the best, independent of the learning algorithm a mutual information-based [33], correlation coefficient [34], and the Chi-square test (Chi2) [7].

#### 3.5.2. Wrapper Approach

The wrapper approach mainly depends on the performance of the learning algorithm. The chosen feature subsets estimate the model performance. According to the model performance, the algorithm adds or removes features until the optimal feature subset is reached. It is more computationally expensive than the filter approach because it repeats the learning and evaluation process. However, it is considered to be more accurate and efficient than the filter approach. The best feature subset is mainly chosen based on the classifier performance. Sequential feature selection [35] and recursive feature elimination (RFE) [36] is an example of this approach.

#### 3.5.3. Embedded Approach

The third method is the embedded approach. This approach uses both ensemble and hybrid learning to make FS. It works by choosing the best features during the learning process. Selecting the optimum feature subset chosen during the training process takes advantage of enhancing computational cost. Since it depends on a collective decision, its performance is better than the filter and wrapper approach regarding computational cost and classification accuracy. Several techniques developed in terms of embedded FS include tree based (RF) and Relevant Sample-Feature Machine (RSFM).

### 3.6. Splitting Dataset

The PCOS was split into two sets using a stratified sampling method, i.e., a ratio of 80% training set and 20% testing sets and a ratio of 70% training set and 30% testing set. Training sets are used to train and optimize models; the testing sets are used to evaluate models.

### 3.7. Models Optimization and Training

Bayesian Optimization (BO) is used to optimize diffident ML models using training sets and cross-validation.

#### 3.7.1. ML Models

We used different ML models, namely logistic regression (LR) [37], random forest (RF) [38], decision tree (DT), naive Bayes (NB) [39], support vector machine (SVM) [40], k-nearest neighbor (KNN) [41], Xgboost [42], and the Adaboost algorithm [43].

#### 3.7.2. Bayesian Optimization

Hyperparameter optimization techniques aim to find the optimum hyperparameter that gives the best performance on a validation set [44]. It can be represented with the following Equation [44]:

(1) $$x^{★}=\arg\min_{c\in X}f\left(x\right)$$

where $x^{★}$ is the optimum hyperparameter list that will give the best performance, f(x) is the objective that needs to be minimized, such as the error rate evaluated based on the validation set, and *c* represents any value in the *x* domain [44].

Using uniform hyperparameter optimization such as grid search and random search gives enhanced performance over a manual search. It starts with a list of values for each hyperparameter and runs a train-predict-evaluate loop. The problem with this approach is that it is completely uniform and does not consider the previous evaluation. Therefore, it could take significant time to evaluate bad hyperparameters. In contrast, BO considers past performance when building a probability model of the objective function [45].

This model is known as a “surrogate” that could represent (p(Y|X). This model works by finding the next list of hyperparameters that perform best according to the surrogate function.

### 3.8. Stacking Machine Learning

The ensemble model builds on combining decisions from several models to improve the model’s overall performance. This approach enhances performance over a single model [46,47]. Bagging, boosting, and staking are the most popular ensemble techniques. Stacking is an ensemble technique that combines different classifications through a meta-classifier [48]. The base model (base classifiers) is trained on the dataset, after which it meta-learns the features that are out of the base classifiers. Therefore, stacking is considered to be one of the more sophisticated heterogeneous classifiers. The architecture of the stacking model includes two or more base models called base–learning, and level-2 is the meta-learning layer that combined the base model’s prediction. Figure 1 shows the general architecture of the stacking ensemble model.

### 3.9. Evaluating Models

As illustrated in Equations (1)–(4), the models are evaluated using four methods: accuracy, precision, recall, and F-score, where TP indicates true positive, TN indicates true negative, FP indicates false positive, and FN indicates false negative:

(2) $$Accuracy=\frac{TP+TN}{TP+FP+TN+FN}.$$

(3) $$Precision=\frac{\mathrm{TP}}{\mathrm{TP}\;+\;\mathrm{FP}}$$

(4) $$Recall=\frac{TP}{TP+FN}$$

(5) $$\mathrm{F-score}=\frac{2\cdot precision\cdot recall}{precision+recall}$$

Furthermore, the models evaluated by the ROC [49] curve is a graphical representation of the performance of a binary classification model. FPR is shown at different classification thresholds. A positive TPR represents the percentage of positive cases that are correctly classified as positive, while a negative FPR represents a percentage of negative cases that are incorrectly classified as positive; AUC (Area Under the Curve) [49] is a measure used to evaluate the performance of a binary rating model, and measures the area under the receiver operating characteristic (ROC) curve, with different rating thresholds, i.e., TPR versus FPR.

## 4. Experimental Results

### 4.1. Experiment Setup

This section presents and discusses the experimental results. Scikit-learn was used to develop the ML models. Google Colab was used to conduct the experiments. Furthermore, the stacking ML models were compared with different ML models based on various feature selection methods (RFE, tree based, and mutual_info). The performance of the models is recorded with two ratios of 20:80 and 30:70 training and testing sets.

### 4.2. Feature Selection Methods

These experiments investigate the essential features of feature selection methods applied to the PCOS dataset.

#### 4.2.1. Scores of Selected Features by Mutual_Info

**Figure 3:**
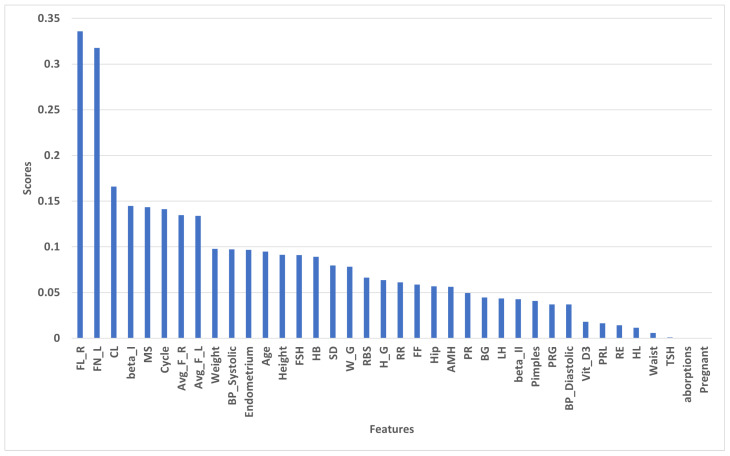
Scores of selected features by mutual_info.

After applying mutual_info to the dataset, the score of each feature is shown in Figure 3. We can see that FL_R has the highest score at 0.33584, and FN_L has the second-highest score at 0.317744. Beta_I, MS, and Cycle have approximately scores of 0.1447437, 0.143511, and 0.1412555, respectively. Vit_D3, PRL, RE, HL, Waist, and TSH have the lowest scores. Aborptions and Pregnant have zero sores. We selected the 30 highest features for applying ML models.

#### 4.2.2. Importance of Selected Features by Tree Based

**Figure 4:**
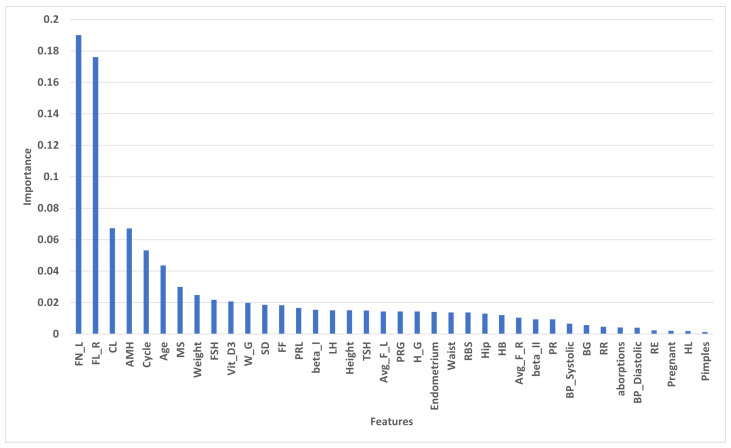
Sores of selected features by based tree.

Figure 4 shows importance of features that are selected features by based tree. FL_R has the highest importance at 0.189997, and FN_L has the second-highest score at 0.176050. CL and AMH have approximate importance of 0.067357 and 0.06720, respectively. RE, Pregnant, HL, and Pimples have the lowest score. We selected the 30 highest features to apply to the ML models.

#### 4.2.3. Ranking of Selected Features by RFE

**Figure 5:**
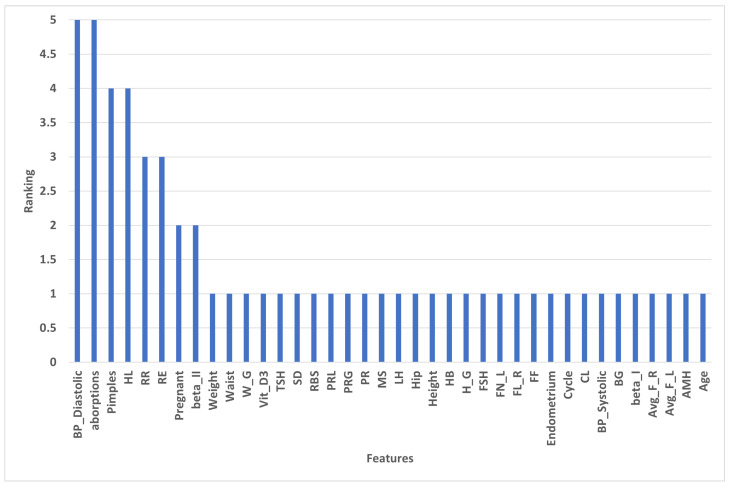
Ranking of the selected features by RFE.

Figure 5 shows REF’s ranking of features; 30 top features have a ranking of 1, such as Age, Weight, Height, BG, PR, HB, Cycle, CL, and MS. The worst features are aborptions and BP_Diastolic, which have a ranking of 5.

### 4.3. Performance of the Classifiers with Selected Features Using Splitting 80:20

**Figure 6:**
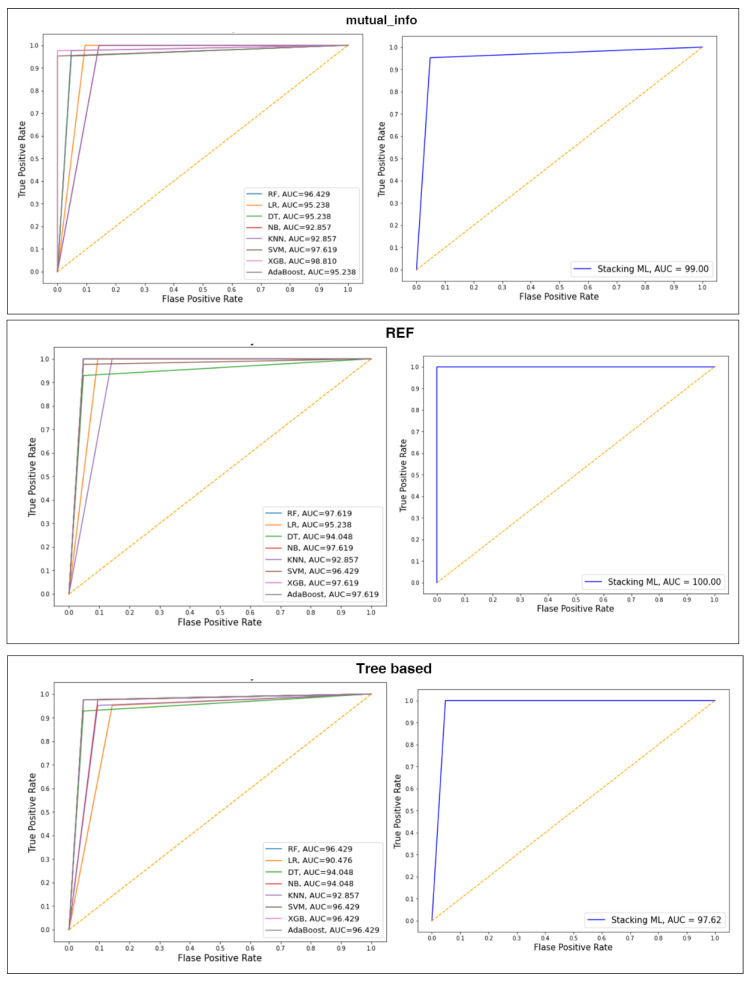
ROC curves of splitting 80:20.

This subsection presents the experimental results of selected features by mutual_info, RFE, and tree based, which are used to train and evaluate the various classifiers with 80:30 splitting. These results are summarized in Table 2. The different classifiers’ AUC values and ROC curves are shown in Figure 6.

Overall, Stacking ML with RFE achieved the highest ACC, PRE, REC, F1, and AUC. For Info_mun, Stacking ML combined the best models to obtain the final prediction and improve performance by 1%, with the highest AUC, ACC, PRE, REC, and F1 of 99, 98.48, 98.41, 98.42, and 98.81, respectively, compared to other models. XGB demonstrated the second-best performance. As we can observe, NB and KNN performed similarly (ACC = 95.24, PRE = 95.56, REC = 95.24 and F1 = 95.14, AUC = 92.86).

For RFE, Stacking ML combined the output of the best models: RF, NB, XGB, and AdaBoost, to obtain the final prediction and improve performance by 1.5 with the highest AUC, ACC, PRE, REC, and F1 of 100, 100, 98.41, 100, and 100, respectively. As we can observe, RF, NB, XGB, and AdaBoost demonstrate the second-best performance (ACC = 98.41, PRE = 98.45, REC = 98.41, and F1 = 98.40, AUC = 97.62). KNN recorded the lowest performance (ACC = 93.65, PRE = 93.99, REC = 93.65 and F1 = 93.72, AUC = 94.05).

For tree based, Stacking ML combined the best models, i.e., RF, SVM, XGB, and AdaBoost, to obtain the final prediction and improve performance by 1.5 with the highest ACC, PRE, REC, F1, and AUC of 97.41, 97.45, 97.41, 97.4, and 97.62, respectively. As we can observe, RF, SVM, XGB, and AdaBoost demonstrate the second-best performance: (ACC = 96.83, PRE = 96.83, REC = 96.83, F1 = 96.83, AUC = 96.43). KNN recorded the lowest performance (ACC = 93.65, PRE = 93.65, REC = 93.65, F1 = 93.65, AUC = 92.86).

### 4.4. Performance of the Classifiers with Selected Features Using Splitting 70:30

**Figure 7:**
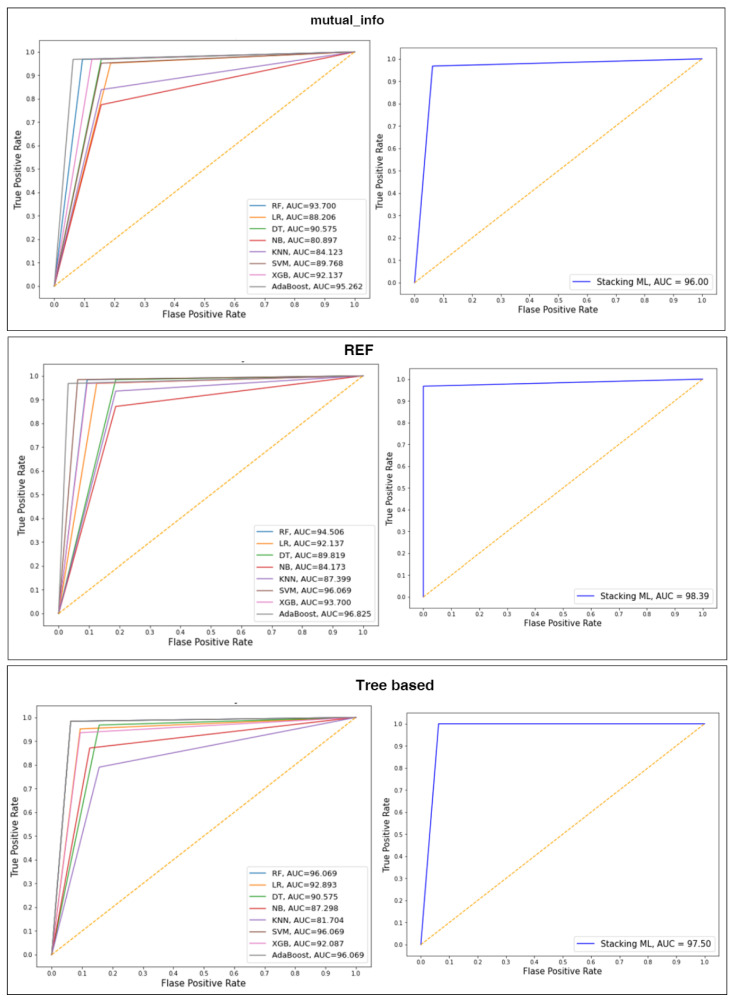
ROC curves of splitting 70:30.

This subsection presents experimental results of selected features by mutual_info, RFE, and tree based are used to train and evaluate the various classifiers with 70:20 splitting. These results are summarized in Table 3. The different classifiers’ AUC values and ROC curves are also shown in Figure 7. Overall, Stacking ML with RFE achieved the highest ACC, PRE, REC, F1, and AUC.

For mutual_info, Stacking ML combined the best models to obtain the final prediction and improve performance by 1%, with the highest ACC, PRE, REC, and F1 of 96.81, 96.81, 96.81, and 96.80, respectively, compared to other models. AdaBoost obtained the second-best performance for ACC, PRE, REC, and F1, i.e., 95.74, 95.74, 95.74, and 95.74, respectively. NB registered the lowest ACC, PRE, REC, and F1, i.e., 79.79, 82.15, 79.79, and 80.24, respectively.

For RFE, Stacking ML combined the best models to obtain the final prediction and improve performance by 1%, with the highest ACC, PRE, REC, and F1 of 98.87, 98.00, 98.87, and 98.89, respectively, compared to other models. AdaBoost obtained the second-best performance for ACC, PRE, REC, and F1, i.e., 96.81, 96.86, 96.81, and 96.82, respectively. NB registered the lowest ACC, PRE, REC, and F1, i.e., 85.11, 85.39, 85.11, and 85.21, respectively.

For tree based, Stacking ML combined the best models to obtain the final prediction and improved performance by 1%, with the highest ACC, PRE, REC, and F1 of 97.81, 97.81, 97.81, and 97.8, respectively, compared to other models. AdaBoost and SVM obtained the second-best performance for ACC, PRE, REC, and F1, i.e., 96.81, 96.81, 96.81, and 96.80, respectively. NB registered the lowest ACC, PRE, REC, and F1, i.e., 87.23, 87.89, 87.23, and 87.40, respectively.

## 5. Discussion

A summary of the experimental results is presented in this section. Additionally, we discuss which model is best for each method of selecting features. The proposed model is also compared to previous studies. Furthermore, model explainability is discussed.

### 5.1. The Best Models

**Figure 8:**
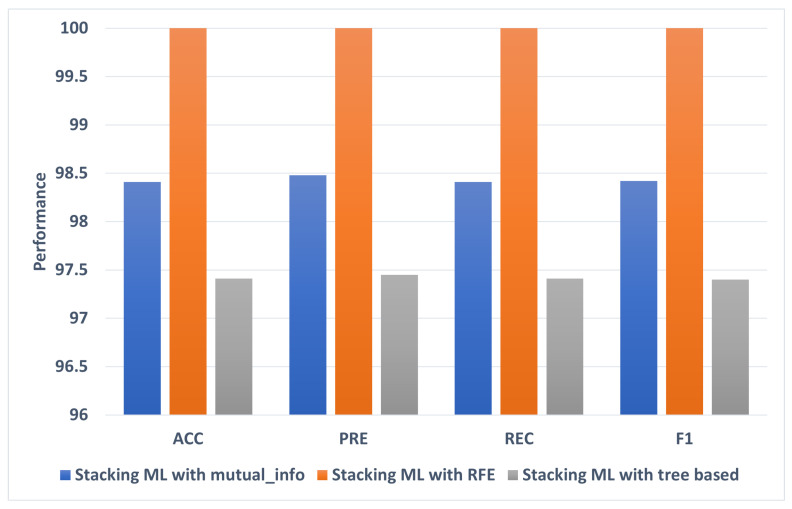
The best models for 80:20 splitting.

Overall, Stacking ML with RFE achieved the highest ACC, PRE, REC, F1, and AUC. Figure 8 shows the best models for each of the 20–80 feature selection methods. Stacking ML with RFE achieves the highest percentages of different evaluation metrics at 100. Stacking ML with tree based recorded has the lowest ACC, PRE, REC, and F1 performance at 97.41, 97.45, 97.41, and 97.4, respectively.

**Figure 9:**
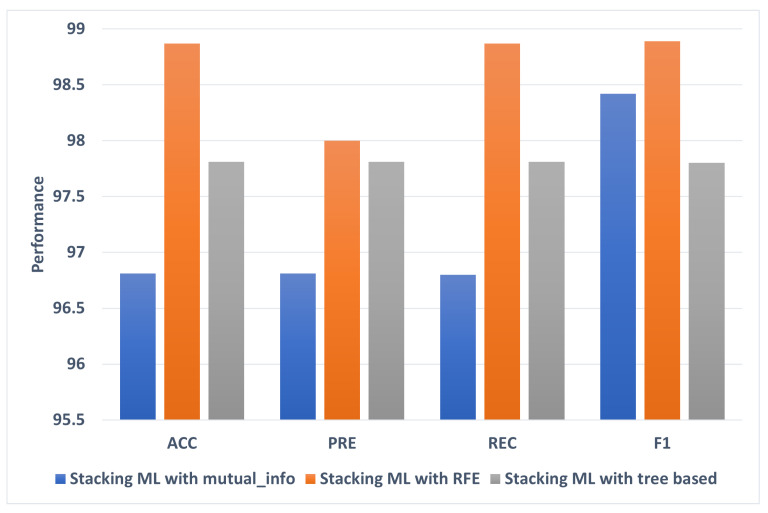
The best models for 70:30 splitting.

Figure 9 shows the best models for each of the 20–80 feature selection methods. Stacking ML with RFE achieved the highest percentages of different evaluation metrics at ACC, PRE, REC, and F1 at 98.87, 98.00, 98.87, and 98.89, respectively. Stacking ML with mutual_info recorded the lowest performance of ACC, PRE, REC, F1, and AUC at 96.81, 96.81, 96.8, and 96.42, respectively.

### 5.2. Comparison with Previous Studies

Table 4 compared previous studies and the proposed model. We can see that our work achieved the highest ACC compared to other studies. In [16], the authors proposed an RFLR hybrid model, applied it with UFS, and achieved an ACC of 91.01. In [17], PCA with RF recorded an ACC of 89.02. In [6], RF with correlation recorded an ACC of 92.4. In [19], SVM with Pearson correlation recorded an ACC of 91.6. In [20], ACC was 91.6 SVM with hybrid feature selection. In [21], RF with chi-square recorded an ACC of 90.9. In [22], DT with Gini importance recorded an ACC of 92.59. In [25], multi-stack of ML recorded an ACC of 98.

### 5.3. Model Explainability

Explainability has two primary levels, i.e., local explainability and global explainability. Global explainability explains the final decision at the level of all data points. It provides casual analysis in terms of global fidelity. It only explained the instance level with the importance of such a level [15]. Local fidelity could explain in terms of all samples. It provides a more accurate explanation. To identify causality and description of the best model (Stacking ML with RFE), in this section, we describe the final decision of the output in terms of global explainability (at the level of a dataset) and local explainability (instance level).

#### 5.3.1. Global Explainability

**Figure 10:**
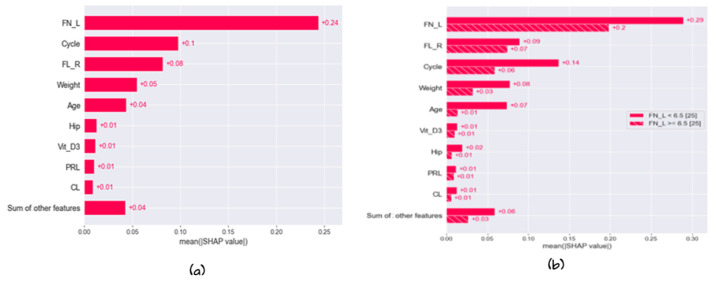
Global explainability of the developed model: (**a**) bar plot (**b**) Cohort plot.

**Figure 11:**
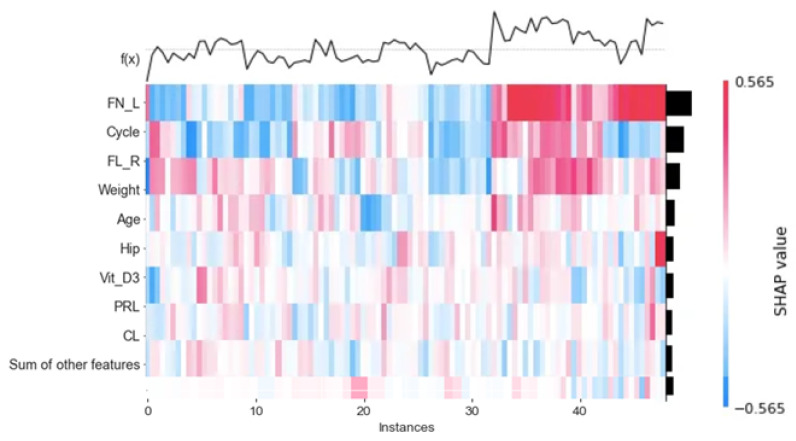
Heatmap of the developed model.

Figure 10a shows the bar plot of the feature importance of each feature with the developed model; in other words, it displays the collective contribution of the features and the less critical features. Figure 10b shows the cohort plot, which divides the total test data into two groups according to the most affected features in all data. As shown in Figure 4, the total data are divided into two main groups according to the number of follicles. The total samples were divided into two groups according to the optimal threshold. Follicles number = 6.5. The bar plot shows that the most affected reason that the instance belongs to the hormonal disorder class is that FL_R (SHAP = 0.09), cycle (SHAP = +0.14), and age (SHAP = 0.07). To provide more information, Figure 5 shows the heat map that shows the importance of the variables in terms of a horizontal bar that shows the rank of the variables from highest to lowest. This importance explains the global interoperability of the developed model. Our developed model depends on FN_L, cycle, and FN_R as the three most important features that affect the overall decision. Sample 32 has a high prediction, which means that FN_L has a significant effect on the prediction. The heatmap in Figure 11 shows the number of instances in the test data in the x_axis, and the curve of F(x) above the plot shows the model prediction for the cases. The observations are also arranged in a way that colors are collected together.

#### 5.3.2. Local Explainability

**Figure 12:**
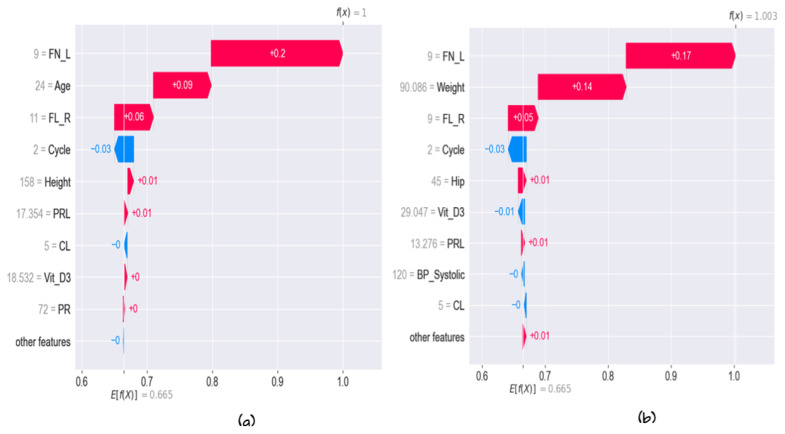
Full water plot for the first and second observation: (**a**) first observation; (**b**) second observation.

**Figure 13:**
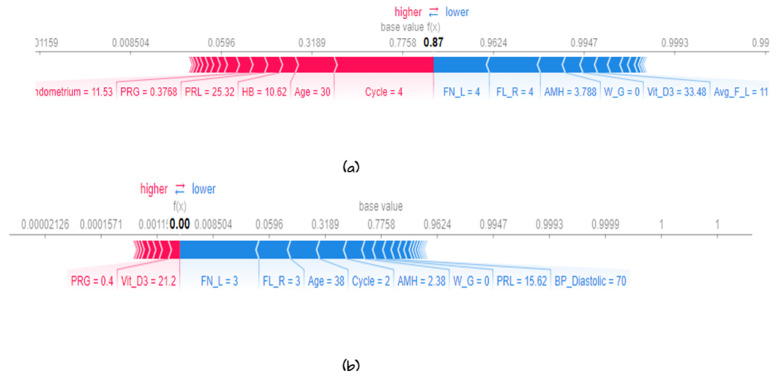
Individual fore plot for several instances according to the developed model (**a**) for observation 1 and (**b**) for observation 2.

**Figure 14:**
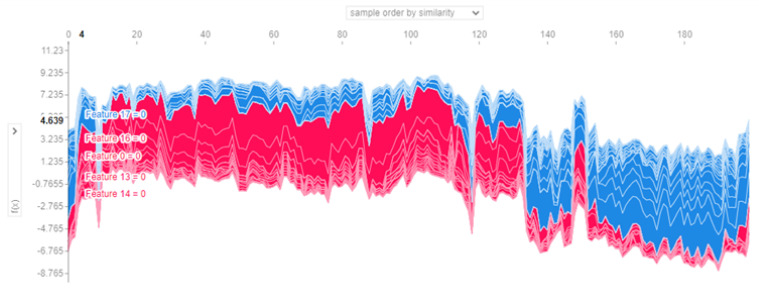
Collective force plot for the developed model.

Many methods are utilized to explain the prediction at the instance level. In this section, we provide several methods to explain the model in terms of instances, such as force plot, water full plot, and summary plot. First, the waterfall plot clarifies why the instance receives the developed value prediction. Figure 12 shows the prediction of the first observation. The prediction for the first observation was 1; this value ranges from (0.665 + 0.2 + 0.09 + 0.6 − 0.03 + 0.01 + 0.01). The values that bedside the variable name refer to the value of the instance feature FN_l = 9, age = 24, etc., and the number in the arrows shows how these features positively or negatively contribute to the decision as shown in Figure 12. The same is true in Figure 12b, which shows the prediction for observation 2 and the values for each feature, and how each feature contributes to the final decision. Second, the force plot explains the key factors. As shown in Figure 13a,b, the plot states that the final prediction of the observation’s higher score led the model to predict 1. In the figure, the bold score was 0.87 for that observation. This means that the observation is highly correlated with class 1. Features with a red color in the horizontal line represent the features that push the model towards a high score, while blue represents features that make the model move towards a low score. Features significantly impacting the final prediction are closer to the dividing boundary between the red and blue areas. All features are sorted from more important to less in the horizontal line in red and blue areas. The same is true for observation 2. The total score was 0. It belongs to class 0 vit_D, and PRG is the feature that pushes the prediction to a high score, and all other features, such as FN_L, FN_R, age, etc., push the prediction to a low score. Figure 14 shows the collective force plot for the developed model. A collective force plot treats the same way as an individual force plot; rotate all samples 90 degrees and add them together.

## 6. Conclusions

The main objective of our paper is to provide an early detection model for PCOS. The early detection of PCOS reduces the possibility of long-term complications. Several ML utilized to build the proposed stacking ensemble ML model. It combines diverse ML models (LR, RF, DT, NB, SVM, KNN, xgboost, and Adaboostare) at the base learner level with RF at the meta-learner level is proposed to improve the performance of a single ML. The following steps apply to build the proposed model: (1) SMOTEENN is applied to the PCOS dataset to solve the class imbalance; (2) Feature selection methods (RFE, tree bases, and mutual info) are applied to select the optimal subset of features; (3) Bayesian optimization finds the optimum hyperparameter that performs best on a validation set; (4) Data are split using two ratios, 70:30 and 80:20; (5) The stacking ensemble model is built with several ML in the base learner level and RF meta learner. The result showed that the Stacking ML with REF feature selection recorded the highest performance at 100 compared to other models with 80:20. It achieved the highest percentages of different evaluation Metrics at ACC, PRE, REC, and F1 at 98.87, 98, 98.87, and 98.89, respectively. To ensure model trust, efficiency, and effectiveness, our research also provides model explanations both at the model level (global explanation) and at the instance level (local explanation).

## Figures and Tables

**Table 1:** Dataset features description details.

Feature Name	Abb	Description
Patient File No.		Patient file number (unique identifier)
Polycystic Ovary Syndrome	PCOS	Class label (determine if the patient has this syndrome or not)
Age	AGE	Patient’s age in years
Weight	WEIGHT	Patient’s weight in KG
Height	HEIGHT	Patient’s height in CM
Body Mass Index	BMI	Body mass index of the patient (height/weight)
Blood Group	BG	Patients belong to which blood group (A+, A−, B+, B−, O+, O−, AB+, AB−)
Pulse Rate	PR	Heartbeat per minute
Respiration Rates	RR	Respiration rates per minute
Hemoglobin	HB	Number of red blood cells in patient’s body
Cycle	CYCLE	Length of the menstrual cycle
Cycle Length	CL	Number of days of a cycle
Marriage Status	MS	Number of years since marriage
Pregnant	P	Pregnant status
No. of Abortions	AB	No. of abortions
I Beta-HCG	BETA_I	Amount of human chorionic gonadotropin
Beta Healthy Singleton Pregnancy	BETA_II	Beta HCG level is indication of 100 mIU/ml about 16 days after ovulation,
Follicle-Stimulating Hormone	FSH	Attributes ranging from 0.3 to 10.0 mIU/mL indicate if are still menstruating or have undergone menopause
Luteinizing Hormone	LH	Chemical agitator that stimulates the reproductive system
Follicle-Stimulating Hormone/ Luteinizing Hormone	FSH/LH	Ratio of FSH and LH
Hip Size	HIP	Size of hip in inches
Waist Size	WAIST	Size of waist in inches
Waist-Hip Ratio	HIP_RATIO	Waist size proportion to hip
Thyroid-Stimulating Hormone	TSH	Amount of TSH in the blood
Anti-Mullerian Hormone	AMH	Plays a key role in developing a baby’s sex organs while in the womb
Prolactin levels	PRL	Prolactin levels in women’s bodies
Vitamin D	VIT_D3	Vitamin D levels
Progesterone Levels	PRG	Progesterone levels
Random Blood Sugar	RBS	Value of random blood sugar (RBS) test
Weight Gain	WG	Test to check if the patient gains weight
Hair Growth	HG	Test to check if a patient has hair growth
Skin Darkening	SD	Test to check the appearance of darkness in skin
Hair Loss	HL	Test to check hair loss
Pimples	PIMPLES	Pimple issues
Fast Food	FF	Check if fast food part of the diet
Reg.Exercise	RE	Check if patient exercises on a regular basis
Blood Pressure Systolic	BP_ SYSTOLIC:	Amount of pressure in the arteries when the heart is contracting
Blood Pressure Diastolic	BP_ Diastolic	Amount of pressure in the arteries while the heart is resting in between heart beats
Follicle No.	FN	Follicle number in the left side

**Table 2:** Performance of the classifiers with selected features using splitting 80:20.

Feature Selection Methods	Models	ACC	PRE	REC	F1
mutual_info	RF	96.83	96.83	96.83	96.83
	LR	96.83	96.97	96.83	96.78
	DT	95.24	95.34	95.24	95.26
	NB	95.24	95.56	95.24	95.14
	KNN	95.24	95.56	95.24	95.14
	SVM	96.83	97.10	96.83	96.86
	XGB	98.12	98.10	98.12	98.12
	AdaBoost	95.24	95.34	95.24	95.26
	Stacking ML	98.41	98.48	98.41	98.42
RFE	RF	98.41	98.45	98.41	98.40
	LR	96.83	96.97	96.83	96.78
	DT	93.65	93.99	93.65	93.72
	NB	98.41	98.45	98.41	98.40
	KNN	95.24	95.56	95.24	95.14
	SVM	96.83	96.83	96.83	96.83
	XGB	98.41	98.45	98.41	98.40
	AdaBoost	98.41	98.45	98.41	98.40
	Stacking ML	100	100	100	100
Tree based	RF	96.83	96.83	96.83	96.83
	LR	92.06	92.02	92.06	92.01
	DT	93.65	93.99	93.65	93.72
	NB	95.24	95.23	95.24	95.21
	KNN	93.65	93.65	93.65	93.65
	SVM	96.83	96.83	96.83	96.83
	XGB	96.83	96.83	96.83	96.83
	AdaBoost	96.83	96.83	96.83	96.83
	Stacking ML	97.41	97.45	97.41	97.4

**Table 3:** Performance of the classifiers with selected features using splitting 70:30.

Feature Selection Methods	Models	ACC	PRE	REC	F1
mutual_info	RF	94.68	94.66	94.68	94.66
	LR	90.43	90.39	90.43	90.30
	DT	92.55	92.58	92.55	92.46
	NB	79.79	82.15	79.79	80.24
	KNN	84.04	85.01	84.04	84.29
	SVM	91.49	91.44	91.49	91.42
	XGB	93.62	93.61	93.62	93.56
	AdaBoost	95.74	95.74	95.74	95.74
	Stacking ML	96.81	96.81	96.81	96.80
RFE	RF	95.74	95.77	95.74	95.71
	LR	93.62	93.61	93.62	93.56
	DT	92.55	92.83	92.55	92.38
	NB	85.11	85.39	85.11	85.21
	KNN	89.36	89.28	89.36	89.27
	SVM	96.81	96.81	96.81	96.80
	XGB	94.68	94.66	94.68	94.66
	AdaBoost	96.81	96.86	96.81	96.82
	Stacking ML	98.87	98.00	98.87	98.89
Tree based	RF	95.81	95.81	95.81	95.80
	LR	93.62	93.62	93.62	93.62
	DT	92.55	92.58	92.55	92.46
	NB	87.23	87.89	87.23	87.40
	KNN	80.85	82.83	80.85	81.25
	SVM	96.81	96.81	96.81	96.80
	XGB	92.55	92.63	92.55	92.58
	AdaBoost	96.81	96.81	96.81	96.80
	Stacking ML	97.81	97.81	97.81	97.8

**Table 4:** Comparison with previous studies.

Papers	Methods	Accuracy
[16]	RFLR with UFS	91.01
[17]	RF with PCA	89.02
[6]	RF with correlation	92.4
[18]	RF	96
[19]	SVM with Pearson correlation	93
[20]	SVM with hybrid feature selection	91.6
[21]	RF with chi square	90.9
[22]	DT with Gini importance	92.59
[25]	multi-stack of ML	98
Our work	Stacking ML with RFE	100

## Data Availability

The direct link in the dataset citations will take you to all of the datasets that were utilized to support the study’s assertions.
